# Comparison of clinical effects between early follicular prolonged GnRH agonist protocol and GnRH antagonist protocol in 3310 cycles: a retrospective study

**DOI:** 10.1186/s12884-022-05295-6

**Published:** 2022-12-15

**Authors:** Juan Gui, Yuan Ni, Qian Liu, Xiaochen Wang, Qingzhen Xie

**Affiliations:** 1 Dept. of Reproductive center, Renmin Hospital of Wuhan University, 238 Jiefang Road, Wuchang District, 430060 Wuhan, China; 2 Assisted Reproduction and Embryogenesis Clinical Research Center of Hubei Province, Wuhan, China

**Keywords:** Controlled ovarian stimulation, Early follicular prolonged GnRH agonist protocol, GnRH antagonist protocol, Assisted reproductive technology, Live birth rate

## Abstract

**Background:**

It is the duty of doctors to choose a safe, simple, economic and effective controlled ovulation stimulation (COS) protocol for the patients. This study aims to compare the clinical effects of the early follicular prolonged GnRH agonist (EFPL) and GnRH antagonist (GnRH-Ant) protocols, hoping to provide some reference for clinicians when choosing COS program.

**Methods:**

A retrospective study included 3310 ovum pick up cycles undergoing assisted reproductive technology during January 2019 to May 2022 in Renmin Hospital of Wuhan University. Propensity Score Matching (PSM) and multivariable logistic regression analysis were used to improve the comparability between the two protocols. Subgroups were divided according to age, body mass index (BMI) and anti-Mullerian hormone (AMH). The live birth rate (LBR) and clinical pregnancy rate (CPR) were the primary outcomes.

**Results:**

After PSM, the endometrial thickness, fresh embryo transplantation rate, chemical pregnancy rate, CPR were significantly higher in EFPL group than that in GnRH-Ant group (*P* < 0.001). The E2, LH, *P* values on trigger day were significantly lower in EFPL group (*P* < 0.001). The cycle cancellation rate was significantly reduced in EFPL group (*P* < 0.001). However, the total amount of Gn and duration of Gn were significantly increased in the EFPL group (*P* < 0.001). Multivariable logistic regression analysis showed that the LBR was significantly higher in EFPL group after matching [OR (95%CI), 1.86 (1.13, 3.05), *P* = 0.02], especially for those with age < 35 years [OR (95%CI), 1.95 (1.14, 3.34), *P* = 0.02], BMI < 24 kg/m^2^ [OR (95%CI), 2.08 (1.14, 3.80), *P* = 0.02], AMH levels ≥ 4.5 ng/ml [OR (95%CI), 4.19 (1.53, 11.43), *P* < 0.01].

**Conclusion:**

EFPL regimen is more suitable to elicit live birth for those young patients with BMI < 24 kg/m^2^ and AMH ≥ 4.5 ng/ml. However, for patients with decreased ovarian reserve or advanced age, EFPL regimen has no advantage over the GnRH-Ant regimen.

## Background

Controlled ovarian stimulation (COS) is a key link of in vitro fertilization and embryo transplantation. It is the duty of doctors to choose a safe, simple, economic and effective COS protocol according to the patient’s age, infertility factors and economic conditions. In order to maximize fresh embryo transplantation and shorten the time to reach gestation, early follicular prolonged GnRH agonist (EFPL) protocol and GnRH antagonist (GnRH-Ant) protocol have become the mainstream regimens in China.

GnRH-Ant regimen has the characteristics of no inhibition of pituitary and ovarian functions which can effectively shorten the time of ovarian stimulation. In addition, agonist can be used as trigger in GnRH-Ant regimen to reduce the incidence of ovarian hyperstimulation syndrome (OHSS). However, the effect of antagonist on endometrial receptivity has been controversial. Several studies suggested a detrimental impact on endometrial receptivity with a significant reduction in the endometrial expression of homeobox A10 during GnRH-Ant cycles compared with GnRH agonist cycles or natural cycles [1, 2]. But Depalo et al. found that GnRH-Ant regimen could achieve live birth rate (LBR) comparable to GnRH agonist regimen [3].

EFPL regimen uses GnRH agonist 3.75 mg to inhibit pituitary function on 2–4 days of menstrual period, then endogenous hormone levels could be completely inhibited within 2 weeks. Gonadotropin (Gn) is started at 28–40 days after successful pituitary downregulation [4, 5]. This regimen was initially used primarily in patients with endometriosis and adenomyosis [6]. After application in the general population, it showed the advantages in improving endometrial receptivity, embryo implantation and clinical pregnancy rates [7, 8]. Therefore, it has been paid more attention by doctors and widely used in clinic [5].

This study aims to compare the clinical effects of EFPL and GnRH-Ant protocols through retrospective data analysis of our reproductive center, hoping to provide some reference for clinicians when choosing COS program.

## Methods

### Study design and patients

This was a retrospective study of women who underwent assisted reproductive technology (ART) at the Reproductive center of Renmin Hospital of Wuhan University between January 2019 and May 2022. Data were collected from the hospital records. Patient’s data included female age, infertility duration and infertility type as well as clinical and laboratory characteristics of ART cycles. The inclusion criteria included that patients were aged < 45 years old underwent fresh cycles; the protocols were EFPL and GnRH-Ant protocols; pregnancy outcomes were followed up at least to gestational 12 week. The cycles for gamete donation program, preimplantation genetic testing, recurrent implantation failure and patients with a history of uterine malformation or intrauterine adhesion or endometrial tuberculosis were excluded from the study. This study was approved by the Ethics Committee of the Renmin Hospital of Wuhan University. The need for individual consent was waived by the committee due to the retrospective character of the study. The primary outcome parameters were LBR and clinical pregnancy rate (CPR). The secondary outcome parameters considered as number of oocytes retrieved, number of 2 pronuclear (2PN) embryos, number of good quality embryos, duration of Gn treatment, amount of Gn administered, OHSS rate, cycle cancellation rate, fresh embryo transfer rate and chemical pregnancy rates. The live birth outcome was followed up until May 2022.

### COS protocols

#### EFPL protocol

Patients were given 3.75 mg long-acting GnRH agonist (Leuprorelin Acetate, ENANTONE, Takeda, Japan) on day 2 to 5 of menstruation. Ultrasound and sex hormone testing were performed 28–30 days after the injection to confirm the successful down-regulation of pituitary with follicle-stimulating hormone (FSH) < 5 U/L, luteinizing hormone (LH) < 5 U/L, estradiol < 50 pmol/L, endometrial thickness < 5 mm, and follicular diameter < 5 mm. Then 87.5–300 IU of recombinant human FSH (rhFSH; Gonal-F, Merck Serono, Switzerland) was started to given according to the patient’s age, body mass index, anti-Mullerian hormone (AMH), antral follicle count. The dose of Gn was adjusted 5 days later according to the patient’s estradiol concentration and ovarian response. After that, patients were returned to the clinic to adjust the dose of Gn every two to three days. When two follicles reached 18 mm in diameter or three dominant follicles reached 17 mm in diameter, moderate human chorionic gonadotropin (hCG, Livzon Pharmaceuticals, China) was administered as a trigger.

#### GnRH-Ant protocol

Patients were given rhFSH (Gonal-F; Merck Serono, Switzerland) 150 to 300 IU/day from day 2 to 3 of menstruation. The dose of Gn was adjusted 3–4 days later according to the patient’s estradiol concentration and ovarian response. An antagonist (cetrorelix; Merck Serono, Switzerland) was added once a day when the average diameter of the dominant follicle was over 14 mm. When two follicles reached 18 mm in diameter or three dominant follicles reached 17 mm in diameter, the final stage of oocyte maturation was induced by moderate hCG (Livzon Pharmaceuticals, China) with or without decapeptyl (0.1 mg) (Ferring International Center SA, Germany).

Transvaginal oocyte retrieval was performed 36 h after the hCG injection. After oocytes retrieved, in vitro fertilization or intracytoplasmic sperm injection was performed according to the condition of the sperms. After culturing for 3 to 5 days, 1 or 2 embryos were selected for transplantation. Then 40 mg progesterone injection (Xianju Pharmaceuticals, Zhejiang, China) and 30 mg oral dydrogesterone tablets (Davutone, Abbott, The Netherlands) were routinely given for luteal support. Blood β-hCG > 10 U/L on day 12 after fresh embryo transplantation indicated chemical pregnancy. Transvaginal ultrasonography was performed 30 days after embryo transfer, and clinical pregnancy was confirmed in the presence of a gestational sac with or without fetal heart activity.

## Statistics

Categorical variables were presented as frequencies or percentages and compared by the chi-square or Fisher’s exact test. Continuous variables were presented as means and standard deviations or medians and interquartile ranges and compared by T-test or Kruskal Wallis Test depending on the distribution. Propensity Score Matching (PSM) was used to balance the baseline and improve the comparability between EFPL group and GnRH-Ant group. The variables in PSM model included female age, BMI, duration of infertility, type of infertility, basal sex hormone (E2, P, FSH, LH), AMH, insemination methods, the number of good quality embryos transferred and the type of embryos transferred. A 1:1 nearest neighbor matching method with caliper (0.05 for ovum pick up cycles; 0.1 for fresh embryo transfer cycles) was used to match data between the two groups. Multivariate logistic regression analysis was performed to compare the live birth rate between the two protocols. Additional analyses were performed after stratification of the participants by age [9] (age < 35 years vs. age ≥ 35 years), BMI (BMI < 24 kg/m^2^ vs. BMI ≥ 24 kg/m^2^) and AMH level [9–11] (AMH < 1.2 ng/ml vs. 1.2ng/ml ≤ AMH < 4.5 ng/ml vs. AMH ≥ 4.5 ng/ml). P < 0.05 was considered to indicate statistical significance. SPSS 26.0 (SPSS Inc. Chicago, IL) was used for the statistical analysis.

## Results

### General outcomes

Before PSM, a total of 3310 fresh cycles were included in this study, including 1537 EFPL regimens and 1773 GnRH-Ant regimens. Significant differences were observed between the two groups in age, BMI, AMH, type of infertility, duration of infertility, cause of infertility, basal FSH, type of fertilization. After 1:1 matching, a total of 800 ovum pick up cycles were analyzed in this study. There were no significant differences in age, BMI, AMH, type of infertility, duration of infertility, cause of infertility, basal FSH, type of fertilization, number of 2PN fertilized embryos, number of transportable embryos, number of good quality embryos and OHSS incidence between the two groups. Compared with the GnRH-Ant group, EFPL group still had significantly higher endometrial thickness, lower E2, LH, *P* values on trigger day, lower cycle cancelation rate and higher fresh embryo transfer rate, but higher duration of Gn and Gn doses (*P* < 0.001). Details were in Table 1.

**Table 1:** Comparison of basic parameters, ovarian stimulation and embryo characteristics between EFPL and GnRH-Ant regimens before and after PS matching

	Before matching			After matching		
Parameter	EFPL	GnRH-Ant	*P*	EFPL	GnRH-Ant	*P*
Number of ovum pick up cycles	1537	1773		400	400	
Age (year)	31.00 (28.00–33.00)	32.00 (29.00–35.00)	< 0.001	30.00 (28.00–33.00)	30.00 (28.00–33.00)	0.67
Primary infertility (%)	59.60 (916/1537)	53.13 (942/1773)	< 0.001	59.75 (239/400)	59.50 (238/400)	0.94
Duration of infertility (year)	3.00 (2.00–5.00)	3.00 (2.00–5.00)	0.33	3.00 (2.00-4.19)	3.00 (2.00–5.00)	0.08
Cause of infertility (%)			< 0.001			0.25
Pelvic and tubal factor	30.32 (466/1537)	22.39 (397/1773)		29.00 (116/400)	26.75 (107/400)	
DOR	1.04 (16/1537)	9.31 (165/1773)		1.50 (6/400)	2.75 (11/400)	
PCOS or ovulation dysfunction	8.85 (136/1537)	8.52 (151/1773)		12.25 (49/400)	11.50 (46/400)	
Endometriosis or adenomyosis	5.53 (85/1537)	1.69 (30/1773)		4.75 (19/400)	2.00 (8/400)	
Male factor	12.23 (188/1537)	9.42 (167/1773)		12.00 (48/400)	10.75 (43/400)	
Complex male and female factors	18.35 (282/1537)	18.89 (335/1773)		19.75 (79/400)	20.50 (82/400)	
Complex female factors	15.29 (235/1537)	23.63 (419/1773)		15.25 (61/400)	19.50 (78/400)	
Others	8.39 (129/1537)	6.15 (109/1773)		5.50 (22/400)	6.25 (25/400)	
BMI (kg/m^2^)	21.70 (19.90–24.00)	22.00 (20.20–24.50)	< 0.01	22.30 (20.20–24.4)	22.00 (20.05–24.20)	0.53
AMH (ng/ml)	3.96 (2.70–5.64)	2.46 (1.20–5.24)	< 0.001	3.94 (2.57–6.38)	3.75 (2.29–6.20)	0.17
Basal E_2_ (pg/ml)	41.92 (32.14–56.26)	42.66 (34.14–55.69)	0.15	43.04 (33.25–55.40)	41.94 (34.34–52.56)	0.60
Basal LH (mIU/ml)	3.97 (2.84–5.54)	3.85 (2.71–5.31)	0.38	4.07 (2.99–5.54)	4.02 (2.88–5.50)	0.74
Basal FSH (mIU/ml)	7.16 (5.89–8.41)	7.74 (6.42–9.48)	< 0.001	7.34 (6.15–8.52)	7.45 (6.20–8.77)	0.19
Basal P (ng/ml)	0.55 (0.37–0.75)	0.55 (0.38–0.76)	0.53	0.53 (0.36–0.73)	0.55 (0.39–0.73)	0.40
E2 on trigger day (pg/ml)	2732.41 (1890.96-3774.40)	2750.94 (1641.90-4260.62)	0.99	2716.15 (1884.29-3685.65)	3188.85 (2172.30-4899.94)	< 0.001
LH on trigger day (mIU/ml)	0.56 (0.31–0.96)	1.73 (0.95–3.12)	< 0.001	0.49 (0.28–0.94)	1.52 (0.82–2.73)	< 0.001
P on trigger day (ng/ml)	0.90 (0.65–1.21)	0.93 (0.65–1.29)	0.05	0.88 (0.58–1.21)	1.02 (0.70–1.42)	< 0.001
Gn dose (IU)	2025.00 (1611.25-2536.25)	2025.00 (1650.00-2512.50)	0.05	2100.00 (1650.00-2625.00)	1950.00 (1579.38-2346.88)	< 0.01
Gn duration (day)	11.00 (10.00–13.00)	10.00 (9.00–11.00)	< 0.001	11.00 (10.00–13.00)	10.00 (9.00–11.00)	< 0.001
Endometrial thickness (mm)	12.20 (10.70–14.00)	11.00 (9.90-12.45)	< 0.001	12.00 (10.80–14.00)	11.00 (10.00-12.15)	< 0.001
Oocytes retrieved (n)	13.00 (9.00–18.00)	10.00 (5.00–17.00)	< 0.001	12.00 (9.00–18.00)	13.00 (8.00–20.00)	0.50
Type of fertilization (%)			< 0.001			0.31
IVF	73.06 (1123/1537)	70.11 (1243/1773)		72.00 (288/400)	69.50 (278/400)	
ICSI	19.45 (299/1537)	26.06 (462/1773)		21.50 (86/400)	25.50 (102/400)	
Rescue ICSI	7.48 (115/1537)	3.84 (68/1773)		6.50 (26/400)	5.00 (20/400)	
Number of 2PN fertilized embryos n	7.00 (4.00–10.00)	6.00 (3.00–10.00)	< 0.001	7.00 (4.00–10.00)	7.00 (4.00–12.00)	0.16
Number of transportable embryos n	4.00 (2.00–7.00)	3.00 (2.00–6.00)	< 0.001	4.00 (3.00–7.00)	4.00 (2.00–7.00)	0.86
Number of good quality embryos n	3.00 (2.00–6.00)	3.00 (1.00–6.00)	< 0.001	4.00 (2.00–7.00)	4.00 (2.00–7.00)	0.20
OHSS (%)	17.50 (269/1537)	14.33 (254/1773)	0.01	22.00 (88/400)	23.25 (93/400)	0.67
Cycle cancellation rate (%)	3.45 (53/1537)	7.16 (127/1773)	< 0.001	2.25 (9/400)	7.25 (29/400)	< 0.01
Fresh embryo transfer rate (%)	62.91 (967/1537)	40.04 (710/1773)	< 0.001	61.50 (246/400)	32.00 (128/400)	< 0.001
Reasons for canceling the fresh embryo transfer (%)			< 0.001			< 0.01
Prevention of OHSS	54.56 (311/570)	39.51 (420/1063)		62.34 (96/154)	45.59 (124/272)	
Embryo factor	9.47 (54/570)	12.98 (138/1063)		7.79 (12/154)	12.13 (33/272)	
Endometrial factor	13.51 (77/570)	13.45 (143/1063)		9.09 (14/154)	11.03 (30/272)	
Elevated *P* value	10.18 (58/570)	18.72 (199/1063)		10.39 (16/154)	19.12 (52/272)	
Hydrosalpinx	1.93 (11/570)	1.03 (11/1063)		1.30 (2/154)	0	
Patient factors or others	10.35 (59/570)	14.30 (152/1063)		9.09 (14/154)	12.13 (33/272)	

Continuous data are presented as median (25%IQR-75%IQR) and discontinuous data are presented as percentage. *IQR* Interquartile range, *EFPL* Early follicular prolonged GnRH agonist, *GnRH-Ant* GnRH antagonist, *DOR* Decreased ovarian reservation, *PCOS* Polycystic ovarian syndrome, *BMI* Body mass index, *AMH* Anti-Mullerian hormone, *E2* Estradiol, *LH* Luteinizing hormone, *FSH* Follicle-stimulating hormone, *P* Progesterone, *Gn* Gonadotropin, *IVF* In vitro fertilization, *ICSI* Intracytoplasmic single sperm injection, *2PN* Two pronuclear, *OHSS* Ovarian hyperstimulation syndrome

Chi-square test and Mann-Whitney U test were used for the comparison between the two groups. The variables in PSM model included female age, BMI, duration of infertility, type of infertility, basal sex hormone (E2, P, FSH, LH), AMH and insemination methods. A 1:1 nearest neighbor matching method with caliper 0.05 was used to match data between the two groups

After 1:1 matching, a total of 332 fresh transfer cycles were analyzed in this study. The basal variations were comparable. There were no significantly difference between the two groups in the number of good embryos transferred, miscarriage rate, heterotopic pregnancy rate. The chemical pregnancy rate (65.06% vs. 48.80%, *P* < 0.01) and CPR (56.02% vs. 38.55%, *P* < 0.01) were significantly higher in EFPL group than that in GnRH-Ant group. The live birth rate was higher in EFPL group but without significance (39.49% vs. 29.03%, *P* = 0.05). The details were in Table 2.

**Table 2:** Comparison of clinical outcomes between EFPL and GnRH-Ant regimens before and after PS matching

	Before matching			After matching		
Parameter	EFPL	GnRH-Ant	*P*	EFPL	GnRH-Ant	*P*
Number of fresh embryo transfer cycles	967	710		166	166	
Number of embryos transferred			< 0.001			0.43
1	32.06 (310/967)	17.46 (124/710)		24.70 (41/166)	21.08 (35/166)	
2	67.94 (657/967)	82.54 (586/710)		75.30 (125/166)	78.92 (131/166)	
Type of embryo transferred			< 0.001			0.01
Cleavage embryo	56.15 (543/967)	85.49 (607/710)		63.86 (106/166)	76.51 (127/166)	
Blastocyst	43.85 (424/967)	14.65 (104/710)		36.14 (60/166)	23.49 (39/166)	
Number of good embryos transferred			< 0.001			0.16
0	14.99 (145/967)	9.58 (68/710)		12.65 (21/166)	6.63 (11/166)	
1	34.23 (331/967)	29.01 (206/710)		28.92 (48/166)	28.92 (48/166)	
2	50.78 (491/967)	61.41 (436/710)		58.43 (97/166)	64.46 (107/166)	
Chemical pregnancy rate per cycle	67.94 (657/967)	49.86 (354/710)	< 0.001	65.06 (108/166)	48.80 (81/166)	< 0.01
Clinical pregnancy rate per cycle	58.12 (562/967)	41.27 (293/710)	< 0.001	56.02 (93/166)	38.55 (64/166)	< 0.01
Live birth rate per cycle	41.54 (356/857)	25.90 (166/641)	< 0.001	39.49 (62/157)	29.03 (45/155)	0.05
Twin pregnancy rate	21.35 (76/356)	22.29 (37/166)	0.81	19.35 (12/62)	11.11 (5/45)	0.25
Miscarriage rate	15.73 (87/553)	17.54 (50/285)	0.50	21.11 (19/90)	11.11 (7/63)	0.10
Pregnancy type			0.42			0.89
Intrauterine pregnancy	98.04 (551/562)	96.59 (283/293)		96.77 (90/93)	98.44 (63/64)	
Heterotopic pregnancy rate	1.60 (9/562)	2.73 (8/293)		3.22 (3/93)	1.56 (1/64)	
Simultaneous intrauterine and extrauterine pregnancy	0.36 (2/562)	0.68 (2/293)		0	0	

Data are presented as percentage. *IQR* Interquartile range, *EFPL* Early follicular prolonged GnRH agonist, *GnRH-Ant* GnRH antagonist

Chi-square test was used for comparison of clinical outcomes between the two groups. The variables in PSM model included female age, BMI, duration of infertility, type of infertility, basal sex hormone (E2, P, FSH, LH), AMH, insemination methods, the number of good quality embryos transferred and the type of embryos transferred. A 1:1 nearest neighbor matching method with caliper 0.1 was used to match data between the two groups

### Live birth measured by multivariate logistic regression with stratification analysis

Before and after matching, and after adjusting for potential confounding factors (such as age, BMI, AMH, basal E2, basal FSH, basal LH, basal P, number of good quality embryos transferred), the multivariate logistic regression analysis showed that the EFPL protocol was associated with a higher possibility of having live birth than that of the GnRH-Ant protocol [OR (95%CI), 1.88 (1.28, 2.77)], (*P* = 0.001); [OR (95%CI), 1.86 (1.13, 3.05)], (*P* = 0.02). The details were in Table 3.

**Table 3:** Comparison of live birth rate of EFPL and GnRH-Ant protocols using multivariable logistic regression analysis in subgroup women with different age, BMI and AMH before and after PS matching (the GnRH-Ant protocol as a reference)

	Before matching		After matching	
	Adjusted OR (95% CI)	*P*	Adjusted OR (95% CI)	*P*
Total	1.88 (1.28, 2.77)	0.001	1.86 (1.13, 3.05)	0.02
Age (year)				
< 35	1.95 (1.28, 2.96)	< 0.01	1.95 (1.14, 3.34)	0.02
≥ 35	0.76 (0.18, 3.21)	0.72	0.67 (0.10, 4.50)	0.68
BMI (kg/m^2^)				
< 24	2.37 (1.46, 3.84)	< 0.001	2.08 (1.14, 3.80)	0.02
≥ 24	1.24 (0.63, 2.47)	0.53	1.54 (0.61, 3.85)	0.36
AMH (ng/ml)				
< 1.2	0	1.00	0	1.00
1.2 ≤ AMH < 4.5	1.75 (1.00, 3.04)	0.05	1.81 (0.93, 3.52)	0.08
≥ 4.5	2.68 (1.30, 5.53)	< 0.01	4.19 (1.53, 11.43)	< 0.01

*CI* Confidence interval. Adjusting for confounders of age, BMI, AMH, basal E2, basal FSH, basal LH, basal P, number of good quality embryos transferred. The variables in PSM model included female age, BMI, duration of infertility, type of infertility, basal sex hormone (E2, P, FSH, LH), AMH, insemination methods, the number of good quality embryos transferred and the type of embryos transferred. A 1:1 nearest neighbor matching method with caliper 0.1 was used to match data between the two groups

A further analysis was conducted to find the live birth rate of the EFPL or GnRH-Ant protocols in patients with different characteristics by stratifying the patients according to their age, BMI and AMH levels. After matching, the multivariate logistic regression analysis showed a significantly higher possibility of having live births of age < 35 years [OR (95%CI), 1.95 (1.14, 3.34)], (*P* = 0.02), BMI < 24 kg/m^2^ [OR (95%CI), 2.08 (1.14, 3.80)], (*P* = 0.02) and AMH ≥ 4.5 ng/ml [OR (95%CI), 4.19 (1.53, 11.43)], (*P* < 0.01) in the EFPL group than in the GnRH-Ant group.

## Discussion

The results of this study showed that the EFPL regimen can obtain a significantly higher CPR and LBR in fresh embryo transfer cycles than GnRH-Ant regimen especially for the patients aged < 35 years with BMI < 24 kg/m^2^ and high ovarian reserve (AMH ≥ 4.5 ng/ml). The cycle cancellation rate was significantly decreased and fresh embryo transfer rate was significantly increased in EFPL group, which may shorten the time to gestation. However, whether before or after matching, the total amount of Gn and duration of Gn were significantly increased in the EFPL group which might give rise to the high cost of patients.

The main factors affecting embryo implantation in ART are embryo quality and endometrial receptivity, of which embryo quality accounts for about 1/3 and endometrial receptivity accounts for about 2/3 [12]. Endometrial thickness has been considered as a prognostic factor in assessing endometrial receptivity and embryo transplantation [13, 14]. Previous studies have showed that the positive rate of β-HCG in fresh embryo transfer cycles increased with the thickening of endometrial thickness on the triggering day, and the optimal threshold of endometrial thickness for transplantation was 10 mm on the triggering day [15–17]. Studies also have shown that fully down-regulation with the long-acting GnRH agonist before COS could obtain optimal endometrial thickness, improve endometrial receptivity [18–23] and LBR [24, 25], which is consistent with the results of our study. GnRH agonist appears to have a direct effect in endometrial cells cultures, by enhancing the percentage of apoptotic cells and decreasing the release of pro-mitogenic cytokines such as interleukin 1beta (IL-1b) and vascular endothelial growth factor during the proliferative phase to inhibit endometriosis [26], but increased IL-1b expression in the mid luteal phase of the menstrual cycle to help implantation [27]. A full dose of GnRH agonist could increase the expression of endometrial receptivity markers such as homeobox A10, myeloid ecotropic viral integration site 1 and leukemia inhibitory factor in endometrium [24]. In this study, the endometrial thickness on the triggering day in the EFPL group was significantly higher than that in GnRH-Ant group. The E2 and *P* values on trigger day were significantly reduced in EFPL group which would also improve the receptivity of endometrium. Although the number of good quality embryos transferred was comparable between the two groups, the CPR and LBR were still higher in the EFPL group. It further indicated that the better pregnancy outcome was attributed to the endometrial factors and verified that the EFPL regimen was more suitable for fresh embryo transplantation.

Although the EFPL regimen can improve endometrial receptivity and LBR, the full down-regulation of pituitary may require a higher dose of Gn for ovarian stimulation [28, 29]. The GnRH-Ant protocol avoids excessive pituitary suppression and flare-up side effects, requires a shorter usage duration and lower total dosage of Gn, and reduces the incidence of severe OHSS. Our study showed that although OHSS incidence was not significantly different between the two groups, the total dosage of Gn and duration of Gn were significantly higher in EFPL group than GnRH-Ant group, which was in consistent with previous studies [30, 31]. This means EFPL protocol would increase the economic burden for infertile couples. It should not be recommended to all type of infertile couples. Xia et al. [30] found that the EFPL protocol had a higher clinical pregnancy rate in normal ovarian responders (age < 35 years and AMH > 1.2 ng/ml) with the fresh transfer cycles than the GnRH-Ant protocol. The results of this study are consistent with our findings. According to our result, only patients aged < 35 years with low BMI and high AMH had received more benefits from EFPL protocol than GnRH-Ant protocol, so we might recommend the EFPL protocol for this population. However, the outcomes of stratified analysis from Chen et al. [31] were different from ours. They found that for those with AMH levels between 3 ng/ml and 6 ng/ml, with BMI ≥ 24 kg/m^2^ and were aged ≥ 30 years old, and for those women with BMI < 24 kg/m^2^ and were aged ≥ 30 years whose AMH levels were ≤ 3 ng/ml, the EFPL protocol was more likely to elicit live births [OR (95%CI), 2.13(1.19,3.80)], [OR (95%CI), 1.41(1.05,1.91)]. These differences might come from the different stratification methods in the studies.

Female age is an independent risk factor for embryo quality [32, 33]. With increasing age, the number and quality of oocytes are significantly reduced [34]. Li Fei et al. analyzed clinical data from 45,912 in vitro fertilization/intracytoplasmic sperm injection cycles and found that EFPL was the most effective protocol than GnRH-Ant protocol for young poor ovarian responders. However, there were no differences in the implantation rates, CPR, or LBR in older patients [5]. Our study also found that for patients with decreased ovarian reserve or age ≥ 35 years old, there was no difference in LBR between the two regimens. The poor embryo quality counterbalances the advantage of good endometrial receptivity in this population.

There are some limitations in the present study. Firstly, this study was a retrospective analysis and the research subjects were patients in one reproductive center, which might be likely to cause some deviations in the results. Secondly, the present study only focused on the effects of fresh embryo transfer and did not examine frozen embryo transfer. Last but not least, due to the limited sample size, we did not continue to conduct stratified analysis on all the causes of infertility.

## Conclusion

The EFPL regimen can effectively improve endometrial receptivity, facilitate embryo implantation, improve the CPR and LBR in fresh cycles, and reduce the time to reach gestation without increasing the OHSS incidence. However, the increased cost might limit its wide application. For patients aged < 35 years with BMI < 24 kg/m^2^ and AMH ≥ 4.5 ng/ml, EFPL regimen is recommended as the first choice to achieve higher LBR in the fresh cycles. However, for patients with decreased ovarian reserve or advanced age, EFPL regimen could not improve the pregnancy rate but increase the amount and duration of Gn. Thus, GnRH-Ant regimen might be more suitable for these patients.

## Data Availability

The data that support the findings of this study are available from Electronic medical records of Renmin hospital of Wuhan University but restrictions apply to the availability of these data, which were used under license for the current study, and so are not publicly available. Data are however available from the corresponding author upon reasonable request and with permission of Renmin hospital of Wuhan University.

## Abbreviations

AMH: Anti-Mullerian hormone
ART: Assisted reproductive technology
COS: Controlled ovarian stimulation
CPR: Clinical pregnancy rate
EFPL: Early follicular prolonged GnRH agonist
FSH: Follicle-stimulating hormone
Gn: Gonadotropin
GnRH-Ant: GnRH-antagonist
hCG: Human chorionic gonadotropin
LBR: Live birth rate
LH: Luteinizing hormone
OHSS: Ovarian hyperstimulation syndrome
rhFSH: Recombinant human follicle stimulating hormone.
